# Repressive Interactions Between Transcription Factors Separate Different Embryonic Ectodermal Domains

**DOI:** 10.3389/fcell.2022.786052

**Published:** 2022-02-07

**Authors:** Steven L. Klein, Andre L. P. Tavares, Meredith Peterson, Charles H. Sullivan, Sally A. Moody

**Affiliations:** ^1^ Department of Anatomy and Cell Biology, The George Washington University School of Medicine and Health Sciences, Washington, D.C., DC, United States; ^2^ Department of Biology, State College, Penn State University, University Park, PA, United States; ^3^ Department of Biology, Grinnell College, Grinnell, IA, United States

**Keywords:** neural plate, neural border zone, neural crest, placode, epidermis, foxd4

## Abstract

The embryonic ectoderm is composed of four domains: neural plate, neural crest, pre-placodal region (PPR) and epidermis. Their formation is initiated during early gastrulation by dorsal-ventral and anterior-posterior gradients of signaling factors that first divide the embryonic ectoderm into neural and non-neural domains. Next, the neural crest and PPR domains arise, either *via* differential competence of the neural and non-neural ectoderm (binary competence model) or *via* interactions between the neural and non-neural ectoderm tissues to produce an intermediate neural border zone (NB) (border state model) that subsequently separates into neural crest and PPR. Many previous gain- and loss-of-function experiments demonstrate that numerous TFs are expressed in initially overlapping zones that gradually resolve into patterns that by late neurula stages are characteristic of each of the four domains. Several of these studies suggested that this is accomplished by a combination of repressive TF interactions and competence to respond to local signals. In this study, we ectopically expressed TFs that at neural plate stages are characteristic of one domain in a different domain to test whether they act cell autonomously as repressors. We found that almost all tested TFs caused reduced expression of the other TFs. At gastrulation these effects were strictly within the lineage-labeled cells, indicating that the effects were cell autonomous, i.e., due to TF interactions within individual cells. Analysis of previously published single cell RNAseq datasets showed that at the end of gastrulation, and continuing to neural tube closure stages, many ectodermal cells express TFs characteristic of more than one neural plate stage domain, indicating that different TFs have the opportunity to interact within the same cell. At neurula stages repression was observed both in the lineage-labeled cells and in adjacent cells not bearing detectable lineage label, suggesting that cell-to-cell signaling has begun to contribute to the separation of the domains. Together, these observations directly demonstrate previous suggestions in the literature that the segregation of embryonic ectodermal domains initially involves cell autonomous, repressive TF interactions within an individual cell followed by the subsequent advent of non-cell autonomous signaling to neighbors.

## 1 Introduction

Shortly after gastrulation is completed, the vertebrate embryonic ectoderm is composed of four distinct domains with different fates. The neural plate (NP) will become the brain and spinal cord, the neural crest (NC) will give rise to most of the peripheral nervous system as well as some non-neural tissues, the pre-placodal region (PPR) will contribute to the cranial sensory organs and sensory ganglia, and the epidermis (Epi) will become the skin and its appendages. The process by which these domains arise is believed to involve two main steps: at gastrula stages the embryonic ectoderm is separated into neural and non-neural domains by ventral-to-dorsal (or lateral-to-medial, depending on the animal) gradients of Wnt and BMP signaling, and subsequently the NC and PPR arise at the border between them (reviewed in Stuhlmiller and Garcia-Castro, 2012; Saint-Jeannet and Moody, 2014; Schlosser, 2014; Schlosser et al., 2014; Moody and LaMantia, 2015; Streit, 2018; Seal and Monsoro-Burq, 2020; Thawani and Groves, 2020; Schlosser, 2021). By late neural plate stages, each of the four domains is characterized by a distinct suite of transcription factors (TFs) that are thought to impose domain-specific identity (reviewed in Grocott et al., 2012; Milet and Monsoro-Burq, 2012; Moody and LaMantia, 2015; Streit, 2018; Seal and Monsoro-Burq, 2020; Thawani and Groves, 2020).

Two models have been proposed for how the NC and PPR domains segregate. The “binary competence” model posits that due to the expression of different combinations of TFs and region-specific signals, the lateral border of the neural ectoderm becomes competent to give rise to NC and the medial border of the non-neural ectoderm becomes competent to give rise to the PPR (Ahrens and Schlosser, 2005; Schlosser, 2008; Pieper et al., 2012; Schlosser, 2021). The “border state” model posits that interactions between the neural and non-neural ectoderm produce an intermediate neural border zone (NB) that contains common precursors of both NC and PPR, and their domains subsequently separate *via* differential responses to signals from the underlying tissues and the expression of TFs that are enriched in either the NC or PPR by late neural plate stages (reviewed in Moody and LaMantia, 2015; Seal and Monsoro-Burq, 2020; Thawani and Groves, 2020; Schlosser 2021). This idea is supported by transcriptomic analyses of dissected pieces of ectoderm in frog and chick that showed that at first TFs characteristic of dorsal/midline ectoderm broadly overlap with TFs characteristic of ventral/lateral ectoderm, which by the end of gastrulation resolves into regionally-distinct transcriptional signatures (Hintze et al., 2017; Plouhinec et al., 2017; Trevers et al., 2018). By late neural plate/neurula stages these signatures become more distinct with the expression of TFs that are thought to specify a particular domain. Thus, the acquisition of distinct NP, NC, PPR and Epi fates appears to be a gradual process that involves, at least in part, TF interactions that eventually segregate domains.

**FIGURE 1:**
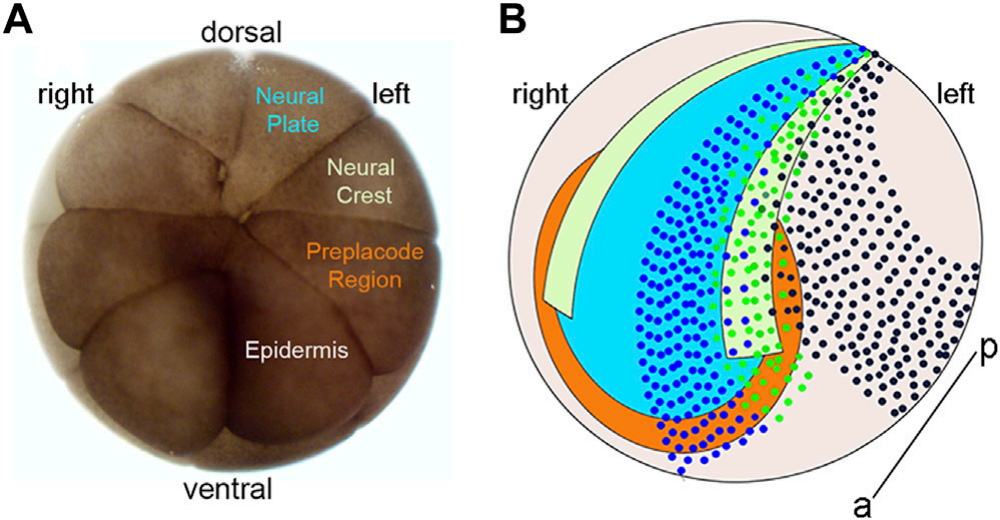
Blastomere fate map and location of clones at neural plate stages. **(A)** Animal view of a 16-cell *Xenopus laevis* embryo indicating the major precursors of the neural plate (blue), neural crest (light green), pre-placode region (PPR; orange) and epidermis (light brown) on the embryo’s left side. **(B)** Cartoon of the ectodermal domains at the neural plate stage with dorsal to the top, anterior to the front, and the anterior-posterior axis (a–p) indicated by a line. Dark blue dots indicate a clone of cells derived from a left blastomere injection that occupies the left neural plate (blue) and left anterior PPR (orange). Green dots indicate a clone of cells derived from a left blastomere injection that occupies the left neural plate border including the left neural crest (light green) and left posterior PPR (orange). Dark brown dots indicate a clone of cells derived from a left blastomere injection that occupies the left dorso-lateral epidermis (light brown).

Consistent with these transcriptomic analyses, lipophilic dye tracing of small groups of cells (Streit, 2002; Ezin et al., 2009; Pieper et al., 2011) suggested that the NB is comprised of a mixture of cells that initially are competent to give rise to cells typical of all four neural plate stage domains. Supporting this idea, analysis of TF protein expression at the single cell level found that a subset of cells in the NB expressed TFs characteristic of more than one neural plate stage domain (Roellig et al., 2017). By experimentally manipulating the levels of Sox2 (used as a marker of NP) and Pax7 (used as a marker of NC), these authors suggested the possibility that within a single cell there is competition between TFs that is repressive in nature and ultimately determines the cell’s domain-specific fate. Building upon this work, we asked whether TFs that are enriched in a particular domain at neural plate stages, so-called “landmark” genes (Plouhinec et al., 2017), repress TFs that are enriched in a different domain by taking advantage of the *Xenopus* 16-cell stage fate map (Moody, 1987) to ectopically express the TFs (Figure 1). In nearly every case we found that ectopic expression of TFs enriched in a specific domain at neural plate stages reduced the expression of TFs characteristic of the other three domains.

To assess whether these effects were cell autonomous, we lineage traced the cells that ectopically expressed the exogenous TF. We found that at gastrula stages only the cells carrying the lineage tracer showed reduced TF expression, whereas at neural plate stages reduced TF expression often was additionally observed in cells adjacent to the labeled clone, suggesting that cell-to-cell signaling likely had begun to contribute to segregating the domains. The consistent pattern of mutually reduced expression regardless of the domain or the TF requires that TFs characteristic of more than one domain be expressed in a single cell, as indicated by the protein expression data of Roellig et al. (2017). Since all previous transcriptomic studies were accomplished on bulk RNA preparations of microdissected ectodermal pieces or explants, instead we analyzed a published single-cell RNAseq dataset at the end of gastrulation and at neural tube closure (Briggs et al., 2018). At both stages we detected numerous cells that expressed TFs characteristic of more than one neural plate stage domain. Together, these data support the idea that the segregation of the four ectodermal domains involves mutual repression between TFs characteristic of more than one neural plate stage domain at the single cell level, and later likely includes signaling between cells.

## 2 Materials and Methods

### 2.1 Obtaining Embryos and Microinjections

Fertilized *Xenopus laevis* eggs were obtained by gonadotropin-induced natural mating of wild type, outbred adult frogs as previously described (Moody, 2018a). Embryos were selected at the 2-cell stage if the first cleavage furrow bisected the lightly pigmented region of the animal hemisphere to accurately identify the dorsal-ventral axis (Klein, 1987; Miyata et al., 1987). When these selected embryos reached the 16-cell stage, one animal blastomere that is the major precursor of one of the ectodermal domains (Figure 1; Moody, 1987) was microinjected with 1 nL of a solution containing 100 pg of TF mRNA and 100 pg of lineage tracer mRNA, according to standard methods (Moody, 2018b). This amount of TF mRNA injected was the lowest of the levels reported in previous studies (cited in Section 2.2) that characterized these TFs to alter gene expression.

### 2.2 *In vitro* Synthesis of mRNAs and Antisense RNA Probes

5′capped and polyadenylated mRNAs encoding TFs expressed by cells in the neural plate (*foxd4l1.1*; Sullivan et al., 2001), neural crest (*foxd3*, Sasai et al., 2001; *msx1*, Suzuki et al., 1997; Tribulo et al., 2003; Monsoro-Burq et al., 2005; *zic1*, *zic2*, and *zic3*, Nakata et al., 1997, Nakata et al., 1998), PPR (*six1;* Brugmann et al., 2004), or epidermis (*dlx5*, Papalopulu and Kintner, 1993; Luo et al., 2001), as well as a nucleus-localized *β-galactosidase* (*nβgal*) as a lineage tracer, were synthesized *in vitro* (mMessage mMachine kit, ThermoFisher). Antisense RNA probes for *in situ* hybridization (ISH) were synthesized *in vitro* (MEGAscript kit; ThermoFisher) as previously described (Yan et al., 2009).

### 2.3 Fixation, Histochemistry and *in situ* Hybridization

**FIGURE 2:**
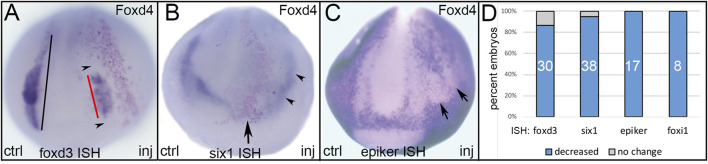
The effects of expressing an NP-enriched transcription factor, Foxd4, in ectopic domains. **(A)** Ectopic expression of Foxd4 (NP TF) in a neural crest precursor blastomere showed reduced size of the *foxd3* neural crest domain (red bar) on the injected side of the embryo. Compare to the length of the *foxd3* expression domain on the control side (black bar). Note that *foxd3* is reduced both in areas occupied by lineage-labeled cells (red dots) as well as areas adjacent to these cells (black arrowheads). ctrl, control side; inj, injected side, anterior view with dorsal to the top. **(B)** Ectopic expression of Foxd4 (NP TF) at the anterior dorsal midline (red dots) eliminated *six1* expression in the anterior PPR (arrow), and reduced expression in the posterior PPR (black arrowheads) adjacent to the lineage-labeled cells. Anterior view with dorsal to the top. **(C)** Ectopic expression of Foxd4 (NP TF) in a lateral position (red dots) eliminated the expression of *epidermis-specific keratin* (*epiker*) in the lateral epidermis (arrows). Anterior view with dorsal to the top. **(D)** The percentage of embryos in which ectopic Foxd4 reduced expression of neural crest (*foxd3*), PPR (*six1*) or epidermis (*epiker*, *foxi1*) genes. Numbers within the bars denote sample size.

**FIGURE 3:**
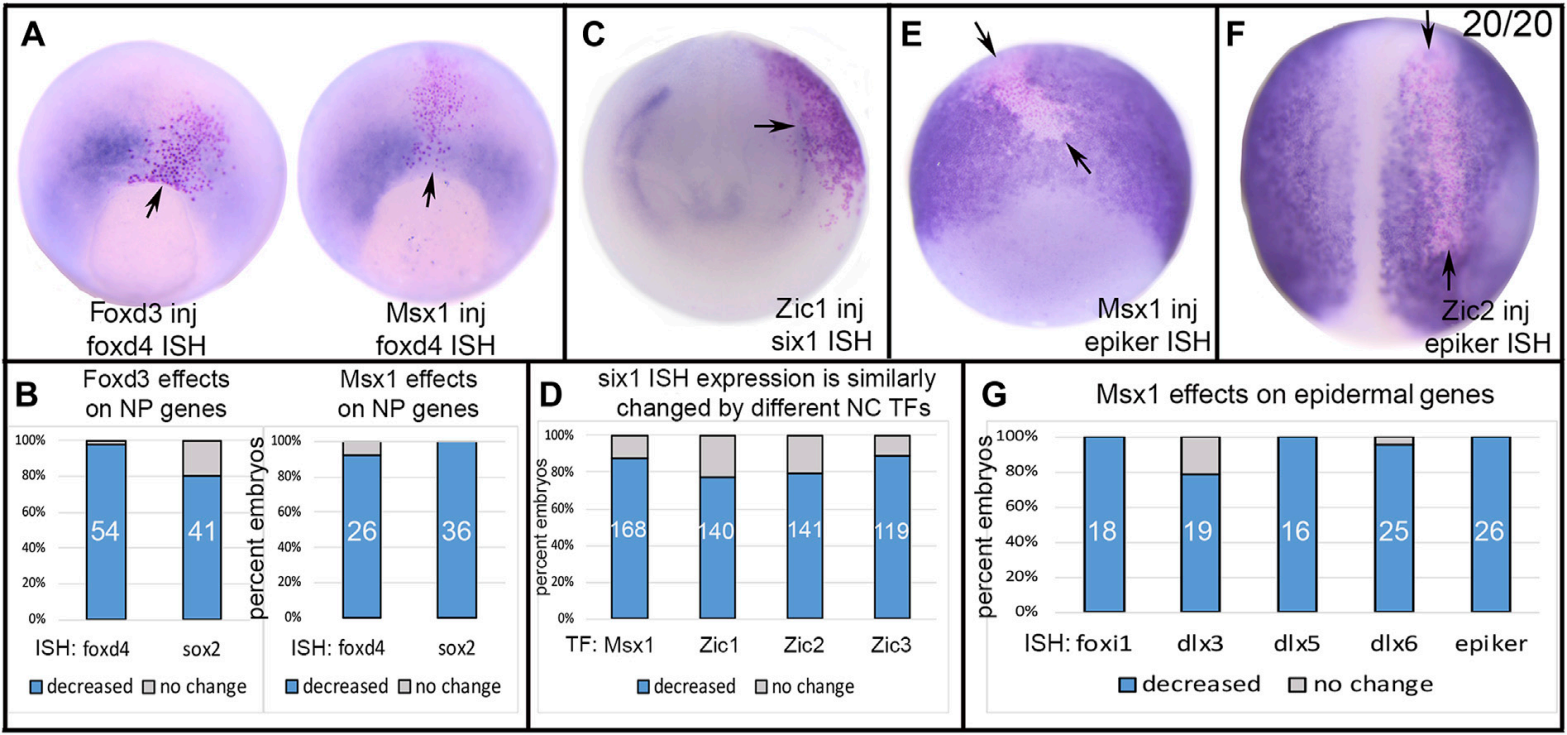
The effects of expressing NC-enriched transcription factors in ectopic domains. **(A)** Ectopic expression of Foxd3 or Msx1(NC TFs) in a neural plate precursor blastomere resulted in reduced expression of *foxd4* (NP TF) only in the region of the lineage-labeled cells (red dots, arrow). Vegetal views at midgastrula (st 11.5) with dorsal to the top. **(B)** The percentage of embryos in which the neural plate expression of *foxd4* or *sox2* were reduced by ectopic expression of either Foxd3 or Msx1. Numbers within the bars denote sample size. **(C)** Ectopic expression of Zic1 (NC TF) in a placode precursor blastomere reduced the PPR expression of *six1* (arrow) in cells expressing Zic1 (red dots). Anterior view with dorsal to the top. **(D)** The percentage of embryos in which the PPR expression of *six1* was reduced by ectopic expression of four different NC TFs. Numbers within the bars denote sample size. **(E)** Ectopic expression of Msx1 (NC TF) in an epidermis precursor blastomere reduced the Epi expression of *epiker* (between arrows) only in cells ectopically expressing Msx1 (red dots). Ventral view of gastrula stage with dorsal to the top. **(F)** Ectopic expression of Zic2 (NC TF) in an epidermis precursor blastomere reduced the Epi expression of *epiker* (between arrows) only in cells ectopically expressing Zic2 (red dots). Dorsal view of neurula stage with anterior to the top. **(G)** The percentage of embryos in which the expression of several Epi genes was reduced by ectopic expression of Msx1. Numbers within the bars denote sample size.

**FIGURE 4:**
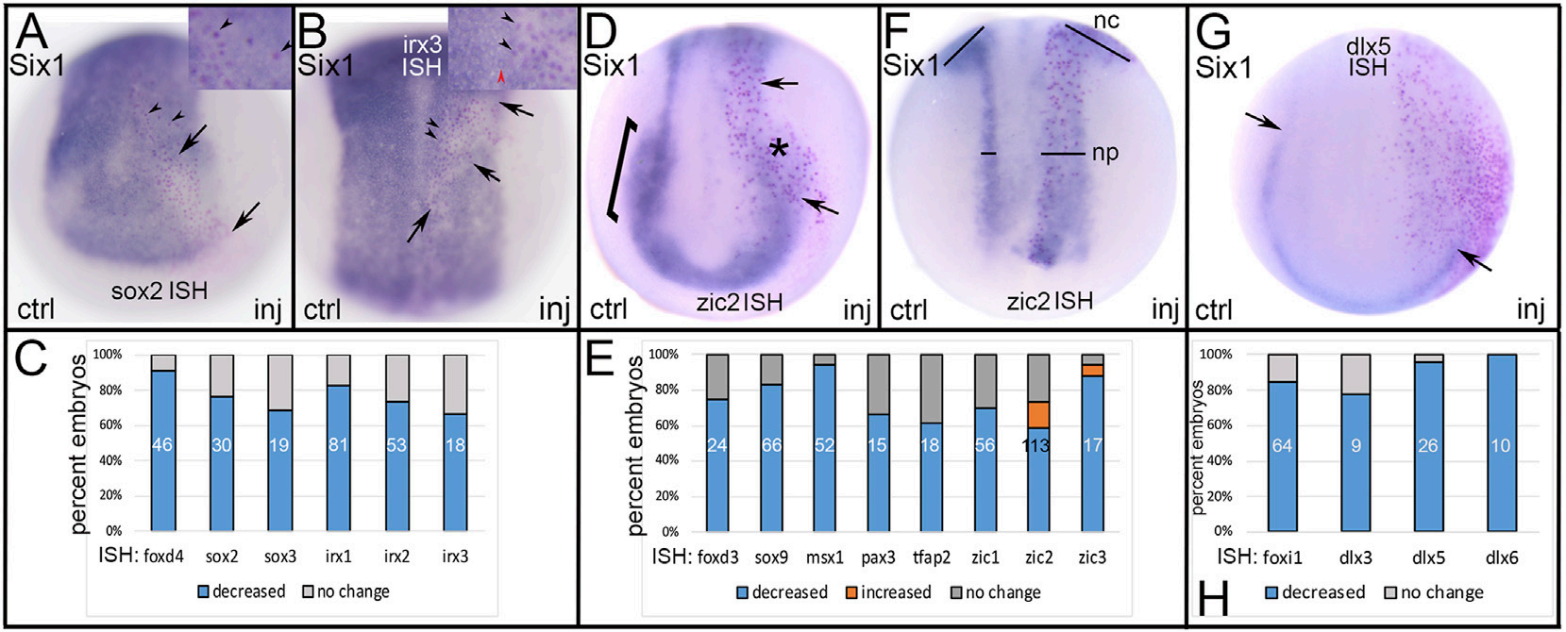
The effects of expressing a PPR-enriched transcription factor, Six1, in ectopic domains. **(A)** Ectopic expression of Six1 (PPR TF) in a neural plate precursor blastomere (red dots between arrows) showed reduced expression of *sox2* (NP TF). Arrowheads in low magnification image and inset indicate Six1-expressing cells containing a red lineage-tagged nucleus surrounded by clear cytoplasm denoting reduced *sox2* expression. In this case, the effect was cell autonomous. ctrl, control side; inj, injected side, anterior view with dorsal to the top. **(B)** Ectopic expression of Six1 (PPR TF) in a neural plate precursor blastomere (red dots between arrows) showed reduced expression of *irx3* (NP TF). Black arrowheads in low magnification image and inset indicate Six1-expressing cells containing a red lineage-tagged nucleus surrounded by clear cytoplasm denoting reduced *irx3* expression. In this case, the effect was cell autonomous. Red arrowhead in inset indicates a cell that does not ectopically express Six1 (clear nucleus) and expresses normal levels of *irx3* (dark blue), for comparison. Dorsal view with anterior to the top. **(C)** The percentage of embryos in which the expression of several NP-enriched genes were reduced by ectopic expression of Six1. Numbers within the bars denote sample size. **(D)** Ectopic expression of Six1 (PPR TF) in a neural crest precursor blastomere (red dots between arrows) showed reduced expression of *zic2* in both the neural plate (arrows) and neural crest (*) domains. The NC domain of *zic2* on the control side is indicated by a bracket. Anterior view with dorsal to the top. **(E)** The percentage of embryos in which the expression of several NC-enriched genes was reduced (blue) or expanded (orange) by ectopic expression of Six1. Numbers within the bars denote sample size. **(F)** In a small number of embryos, ectopically expressed Six1 (PPR TF) expanded the neural plate (np) and neural crest (nc) expression domains of *zic2.* Bars compare the widths between control (ctrl) and injected (inj) sides. Dorsal view with anterior to the top. **(G)** Ectopic expression of Six1 (PPR TF) in a lateral precursor blastomere (red dots) reduced expression of *dlx5* (Epi TF) in the epidermis along the border zone. Arrows indicate the posterior limit of the *dlx5* domain on control (ctrl) and injected (inj) sides. Anterior view with dorsal to the top. **(H)** The percentage of embryos in which the expression of several Epi-enriched genes was reduced by ectopic expression of Six1. Numbers within the bars denote sample size.

**FIGURE 5:**
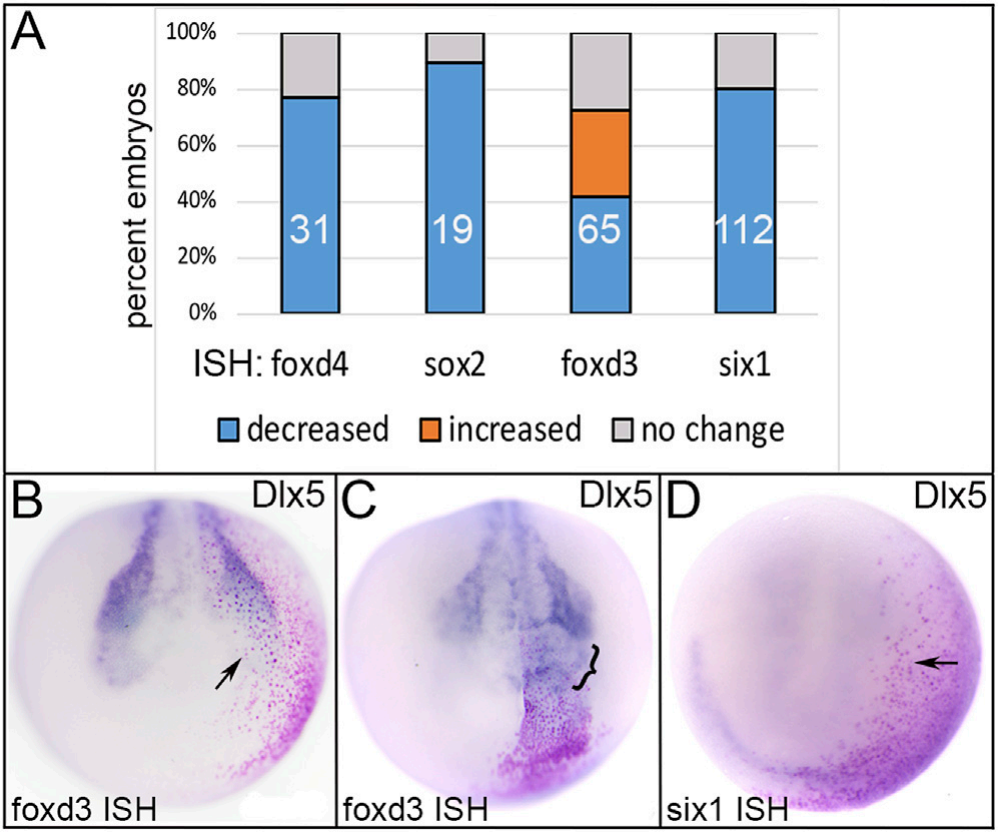
The effects of expressing an Epi-enriched transcription factor, Dlx5, in ectopic domains. **(A)** The percentage of embryos in which ectopically expressed Dlx5 reduced (blue) or expanded (orange) expression of neural plate (*foxd4, sox2*), neural crest (*foxd3*) or PPR (*six1*) genes. Numbers within the bars denote sample size. **(B)** Ectopic expression of Dlx5 (Epi TF) in a neural crest precursor blastomere (red dots) reduced expression of *foxd3* (NC TF; arrow). Anterior view with dorsal to the top. **(C)** Ectopic expression of Dlx5 (Epi TF) in a neural plate blastomere (red dots) broadened the expression of *foxd3* (NC TF; bracket). Anterior view with dorsal to the top. **(D)** Ectopic expression of Dlx5 (Epi TF) in a placode precursor blastomere (red dots) reduced the expression of *six1* (PPR TF; arrow). Anterior view with dorsal to the top.

**FIGURE 6:**
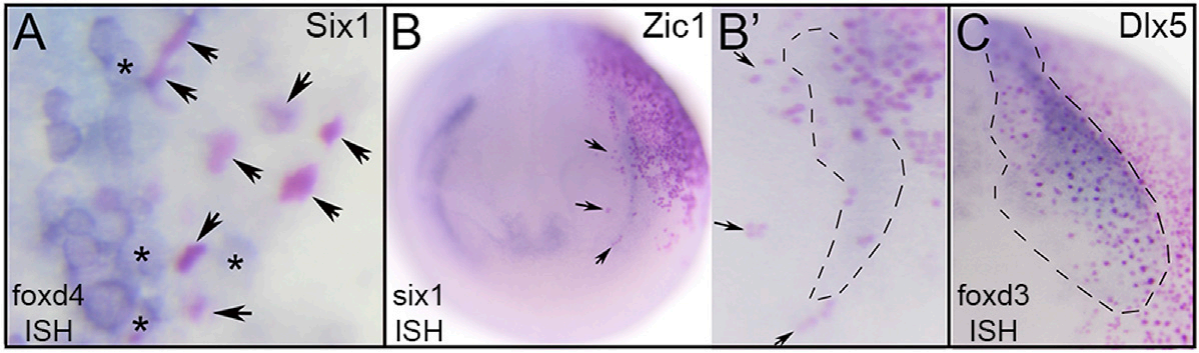
TF effects are cell-autonomous at gastrula stages but likely include signaling to neighbors at neurula stages. **(A)** Dorsal midline of gastrula showing Six1-expressing cells that have lineage-labeled nuclei (arrows, red dots). Each of the lineage-labeled cells showed reduced *foxd4* expression (clear cytoplasm). In contrast, uninjected neighbors (asterisks, clear nuclei) showed high levels of *foxd4* expression (blue cytoplasm). **(B)** Ectopic expression of Zic1 (NC TF) in a placode precursor blastomere (red dots) showing Zic1-expressing cells on right side of neurula stage embryo (red nuclei) overlapping with the reduced *six1* PPR expression domain (blue). Anterior view with dorsal to the top. B′ shows a higher magnification of the *six1* PPR domain on the injected side. For orientation, arrows point to the same cells as in B. Each of the lineage-labeled cells showed reduced *six1* PPR expression, but within the region surrounded by the dashed line, uninjected neighbors also had reduced expression. **(C)** Ectopic expression of Dlx5 (Epi TF) in a neural crest precursor blastomere (red dots) showing Dlx5-expressing cells (red nuclei) within the *foxd3* neural crest expression domain outlined by dashes of a neurula stage embryo. Within that domain, lineage-labeled cells showed reduced *foxd3* neural crest expression, but it also was reduced in adjacent uninjected neighbors. Anterior view with medial to the left and dorsal to top.

Embryos were cultured to gastrula (st 11.5–13) or neurula (st 16–18) stages (Nieuwkoop and Faber, 1994), fixed in 4% paraformaldehyde (in 0.1 M MOPS, 2 mM EGTA Magnesium, 1 mM MgSO_4_, pH 7.4), stained for βGal histochemistry to reveal the cells that received the exogenous mRNA, and processed for *in situ* hybridization (ISH) as previously described (Yan et al., 2009). Each experiment was repeated in 2–5 independent trials with different sets of parents to ensure genetic diversity in the samples. Embryos were scored for gene expression changes, comparing the injected (β-Gal-positive) versus the uninjected side of the same embryo, independently by at least two of the authors, and the values reported are means of their independent scores. For Figures 2–5, only embryos in which the β-Gal-positive cells were within the domain of the assessed gene were included in the analysis. For Figure 6, only embryos in which βGal-positive cells were not within the domain of the assessed gene were included in the numbers presented in Tables 1, 2. As injection controls, only *nβgal* mRNA was injected into a blastomere, and the expression of at least 2 TFs enriched in each domain were analyzed by ISH. In nearly every case, the expression domain on the injected side was the same as that on the uninjected side of the same embryo [NP: *foxd4* (100%, *n* = 11), *sox2* (100%; *n* = 18), *irx1* (100%, *n* = 22); NC: *foxd3* (94.7%, *n* = 19), *sox9* (95.5%, *n* = 22); PPE: *six1* (100%, *n* = 25), *irx1* (100%, *n* = 22), *sox9* (100%, *n* = 22); Epi: *dlx5* (100%, *n* = 22), *foxi* (100%, *n* = 31)]. These controls verify that the observed expression changes reported below were due to the TF mRNAs not the lineage tracer.

**TABLE 1:** The number of cases in which cells distant from the lineage label showed reduced expression at gastrula stages.

mRNA injected	*foxd4* ISH	*sox2* ISH	*msx1* ISH	*foxi1* ISH
	st11-13	st11-13	st11-13	st11-13
*foxd3*	0/3	0/2	No cases	No cases
*msx1*	0/15	0/6	No cases	0/6
*six1*	0/11	No cases	0/5	0/11
*dlx5*	0/3	0/4	No cases	No cases

“No cases” means that the dataset did not contain any embryos with distant, lineage-labeled clones.

**TABLE 2:** The number of cases in which cells distant from the lineage label showed reduced expression at neurula stages.

mRNA injected	*foxd3* ISH	*six1* ISH	*dlx3* ISH	*dlx5* ISH	*dlx6* ISH	*foxi1* ISH
	st16-18	st16-18	st16-18	st16-18	st16-18	st16-18
*foxd4*	27/27 (100%)	14/19 (73.7%)	2/5 (40%)	No cases	3/5 (60%)	6/9 (66.7%)
*msx1*	11/15 (73.3%)	5/18 (27.8%)	0/1	1/11 (9.1%)	2/6 (33.3%)	4/6 (66.7%)
*six1*	No cases	Not done	0/3	0/3	0/6	0/4
*dlx5*	2/9 (22.2%)	7/14 (50%)	Not done	Not done	Not done	Not done

“No cases” means that that the dataset did not contain any embryos with distant, lineage-labeled clones. “Not done” means that we did not perform this experimental combination.

### 2.4 Analysis of Single Cell RNAseq Dataset

We utilized the single cell RNAseq dataset generated by Briggs et al. (2018), which is available online at https://kleintools.hms.harvard.edu/tools/currentDatasetsList_xenopus_v2.html. We extracted data from reference SPRING plots for Stage 13 and Stage 18 embryos. These plots contain K-nearest-neighbor (knn) graphs that are used for visualization of data clusters. In these graphs, each cell is represented as a node that extends edges to other nodes/cells that have a similar expression of genes (Weinreb et al., 2018). The Stage 13 plot contains 8,931 raw cells and the Stage 18 plot contains 12,432 raw cells.

Analyses were performed using a two-step process for cell selection. First, aiming to only analyze cells related to neural plate, neural crest, PPR, and epidermis, cells located in “celltype” clusters, designated based on similar transcriptomic signatures, representing these domains were selected. At stage 13, the selected “celltype”clusters were: “anterior neural plate”, “chordal neural plate”, “ionocyte”, “neural crest”, “non-neural ectoderm”, and “placodal area”. At stage 18, the selected “celltype”clusters were: “adenohypophyseal placode”, “anterior neural tube - fezf1”, “anterior neural tube - nkx2-1/nkx2-4”, “anterior placodal area”, “chordal neural crest”, “chordal neural plate border”, “cranial neural crest”, “epibranchial and lateral line placodes”, “epidermal - aqp3”, “epidermal progenitor - tp63/ctbs”, “epidermal progenitor - tp63/tll2”, “ionocyte”, “olfactory placode”, “otic placode”, “placodal neuron - eya2/neurog1/neurod1”, “posterior neural tube”, “posterior placodal area”, and “trigeminal and profundal placodes”. Next, at each stage all cells within the composite of selected clusters that expressed either foxd4l1.1 | FOXD4L1.1, sox2 | SOX2, msx1 | LOC100125666, foxd3 | FOXD3, zic2 | ZIC2-A, six1 | SIX1, dlx5 | DLL3, or foxi1 | FOXI1E were selected and their expression profiles downloaded using the “SPRING data for selection” tool. Eight transcription factor dataset files per stage containing all genes expressed in the selected cells were generated: *foxd4* (stage 13: 44 cells; stage 18: 200 cells), *sox2* (stage 13: 1,192 cells; stage 18: 1,079 cells), *msx1* (stage 13: 284 cells; stage 18: 529 cells), *foxd3* (stage 13: 4 cells; stage 18: 32 cells), *zic2* (stage 13: 128 cells; stage 18: 437 cells), *six1* (stage 13: 111 cells; stage 18: 188 cells), *dlx5* (stage 13: 233 cells; stage 18: 148 cells), foxi1 (stage 13: 191 cells; stage 18: 121 cells). We then determined the number of single cells expressing at least two (Tables 3, 5) or more (Tables 4, 6, 7) transcription factors. In each table, the number of cells that expressed both the selected transcription factor (each column) and one of the other eight analyzed genes (each row) was tabulated. The bottom row of Tables 3, 5 denotes the number of cells across all selected “celltype” clusters that expressed the selected transcription factor (each column), in other words the pool of all cells in the dataset in the selected “celltype” cluster that expressed that gene. This number did not equal the sum of cells in each column because a single cell can express more than two transcription factors.

**TABLE 3:** The number of single cells co-expressing at least two domain-enriched transcription factors at stage 13.

	*foxd4* ^a^	*sox2* ^a^	*msx1* ^a^	*foxd3* ^a^	*zic2* ^a^	*six1* ^a^	*dlx5* ^a^	*foxi1* ^a^
*foxd4* ^c^	-	23	1	0	0	0	0	0
*sox2* ^c^	31	—	67	1	40	37	68	5
*msx1* ^c^	1	36	—	0	6	6	7	2
*foxd3* ^c^	0	0	0	—	0	0	0	0
*zic2* ^c^	9	373	44	0	—	4	12	1
*six1* ^c^	0	11	6	0	1	—	21	1
*dlx5* ^c^	1	44	25	0	8	28	—	15
*foxi1* ^c^	0	2	4	0	0	0	1	—
Total # of cells^a^	44	1192	284	4	128	111	233	191

a Transcription factor dataset that was queried. Single cells were selected based on the expression of the transcription factor at the top of each column in all selected “celltype” clusters defined in Section 2.4.

b The total number of cells across all selected “celltype” clusters that co-expressed the transcription factor at the top of each column. This number did not equal the sum of cells in each column because a single cell can express more than two transcription factors.

c The transcription factor that was co-expressed with the factor at the top of each column.

**TABLE 4:** The number of single cells co-expressing three or four domain-enriched transcription factors at stage 13.

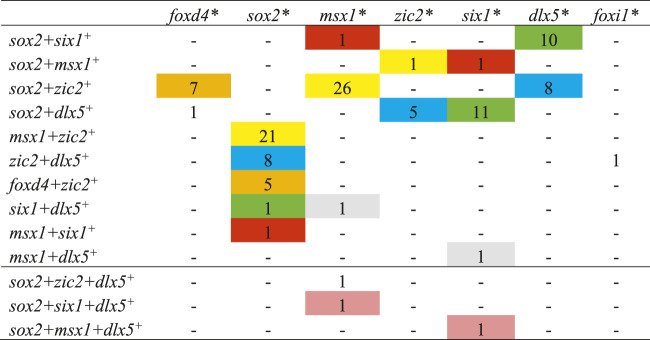	

Cells labeled with the same color co-expressed the same combination of three or four domain-enriched transcription factors.

*Transcription factor dataset that was queried.

+ The transcription factor that was co-expressed with the factor at the top of each column.

**TABLE 5:** The number of single cells co-expressing at least two domain-enriched transcription factors at stage 18.

	*foxd4* ^a^	*sox2* ^a^	*msx1* ^a^	*foxd3* ^a^	*zic2* ^a^	*six1* ^a^	*dlx5* ^a^	*foxi1* ^a^
*foxd4* ^*+*^	-	46	10	6	11	6	3	1
*sox2* ^*+*^	87	-	153	3	358	103	11	13
*msx1* ^*+*^	15	107	-	11	95	15	36	10
*foxd3* ^*+*^	1	4	2	-	3	2	1	0
*zic2* ^*+*^	35	307	97	8	-	7	4	4
*six1* ^*+*^	12	42	8	3	2	-	3	3
*dlx5* ^*+*^	11	79	92	4	12	48	-	20
*foxi1* ^*+*^	11	18	15	3	3	4	1	-
Total # of cells^b^	200	1079	529	32	437	188	148	121

a Transcription factor dataset that was queried. Single cells were selected based on the expression of the transcription factor at the top of each column in all selected “celltype” clusters defined in Section 2.4.

b The total number of cells across all selected “celltype” clusters that co-expressed the transcription factor at the top of each column. This number did not equal the sum of cells in each column because a single cell can express more than two transcription factors.

c The transcription factor that was co-expressed with the factor at the top of each column.

## 3 Results

Many previous studies showed that as the embryonic ectoderm gradually resolves into four distinct domains, numerous TFs are expressed in overlapping patterns that eventually segregate during neurulation into NP, NC, PPR and Epi, each of which characteristically expresses a subset of these TFs (reviewed in Moody and LaMantia, 2015; Seal and Monsoro-Burq, 2020; Thawani and Groves, 2020; Schlosser 2021). It is commonly posed that the overlapping expression domains are sharpened into distinct domains by repressive interactions between these TFs, similar to the interactions between *gap* genes during segmentation in *Drosophila* (reviewed in Jaeger, 2011). To test this possibility, we ectopically expressed TFs that are thought to specify one domain by neural plate stages in a clone of cells that populates a different domain by targeted microinjections of mRNAs into 16-cell blastomere precursors of each domain (Figure 1). Using whole mount ISH, we then assessed the resulting expression patterns of a domain-enriched gene compared to the control, uninjected side of the same embryo. While previous studies focused on *sox2* and *sox3* as NP specifiers, we uniquely focused on the forkhead transcription factor Foxd4l1.1, henceforth referred to as Foxd4, because of its three advantages. It is one of the earliest expressed NP genes (Sullivan et al., 2001; Sherman et al., 2017); it is required for the expression of many other NP genes, including *sox2*-*3* and *irx1-3*; and none of these TFs feedback to regulate it (Yan et al., 2009; Klein and Moody, 2015; Gaur et al., 2016). As in other studies, we ectopically expressed the NC specifier, Foxd3, but additionally ectopically expressed several other TFs that are acknowledged NC specifiers (Msx1, Zic1; Plouhinec et al., 2014; Plouhinec et al., 2017). We also tested other Zic family members (Zic2, Zic3) that are understudied but likewise enriched in the NC domain at neural plate stages and are thought to be functionally redundant with Zic1 (Nakata et al., 1997; Nakata et al., 1998; Sasai et al., 2001; Grocott et al., 2012). We ectopically expressed Six1 to test the effect of an acknowledged PPR specifier that is required for the expression of other PPE genes, including *eya1*, *sox11* and *irx1* (Brugmann et al., 2004; Yan et al., 2015; Riddiford and Schlosser, 2016; Hintze et al., 2017; Sullivan et al., 2019). We ectopically expressed Dlx5 to test the effect of a TF that specifies the dorso-lateral epidermis in *Xenopus* (Luo et al., 2001).

### 3.1 Ectopic Expression of Domain-Enriched TFs Repress TFs Characteristic of Each of the Other Domains

Ectopic expression of Foxd4, a TF that is highly expressed in the early neural ectoderm, acts upstream of several NP genes and can induce their ectopic expression, including *gmnn*, *sox2, sox3, and sox11* (Sullivan et al., 2001; Yan et al., 2009; Gaur et al., 2016), reduced at high frequencies the expression of TFs that at neural plate stages are enriched in either NC, PPR or Epi (Figure 2). We found that ectopic expression of Foxd4 in the dorso-lateral region reduced the NC domain of *foxd3* (Figures 2A,D) and the PPR domain of *six1* (Figures 2B,D). Ectopic expression of Foxd4 in the more ventral ectoderm eliminated expression of an epidermis-specific keratin (*krt12.4*, herein named *epiker*; Figures 2C,D), confirming previous reports (Yan et al., 2009; Gaur et al., 2016), as well as *foxi1* (Figure 2D), which is enriched in the epidermis at late gastrula and neural plate stages (Plouhinec et al., 2017). In no case did ectopic Foxd4 up-regulate the expression of any of the tested NC, PPR or Epi genes.

Foxd3, a key neural crest specifier (Plouhinec et al., 2017; Lukoseviciute et al., 2018), induced the expression of several other neural crest markers in *Xenopus* (Sasai et al., 2001). Msx1 also upregulates *foxd3*, *slug* and *twist* (Tribulo et al., 2003; Monsoro-Burq et al., 2005), and Zic1-3 upregulate *slug* and *twist* (Nakata et al., 1997; Nakata et al., 1998). Herein, we found that Zic2 and Zic3 also increased *foxd3* (54%, *n* = 56 and 67%, *n* = 53, respectively). Ectopic expression of Foxd3 reduced at high frequencies the expression of TFs that at neural plate stages are enriched in either NP, PPR or Epi (Figure 3). Ectopic expression of Foxd3 in the dorsal midline reduced the early NP expression of *foxd4* and *sox2* (Figures 3A,B). Msx1, another NC specifier, had very similar effects on *foxd4* and *sox2* (Figures 3A,B). A previous study also showed that Msx1 repressed the NP expression of *sox3* (Maharana and Schlosser, 2018). We previously demonstrated that ectopically expressing Foxd3 in the PPR reduced the expression domain of *six1* (Brugmann et al., 2004). We expanded this observation by testing whether other TFs enriched in the NC domain at neural plate stages had a similar effect. Indeed, we found that injecting *msx1*, *zic1*, *zic2* or *zic3* mRNAs into blastomeres that contribute to the PPR reduced *six1* expression in the majority of embryos (Figures 3C,D). Previous work demonstrated that Foxd3 represses the expression of *epiker* (Sasai et al., 2001). We expanded this observation and found that ectopic Msx1 and Zic2 also repressed several TFs enriched in the Epi domain at neural plate stages (*foxi1*, *dlx3*, *dlx5*, *dlx6*, *epiker*) (Figures 3E–G). In no case did TFs enriched in the NC at neural plate stages up-regulate the expression of any of the tested NP, PPR or Epi genes.

Six1 is a PPR specifier (Brugmann et al., 2004; Hintze et al., 2017) that upregulates the expression of other PPR genes, including *eya1*, *sox11* and *irx1* (Brugmann et al., 2004; Yan et al., 2015; Riddiford and Schlosser, 2016; Sullivan et al., 2019), but herein we found that it does not alter the PPR expression of other members of the Six family (*six2*, *n* = 48; *six4.1*, *n* = 53). Ectopic expression of Six1 frequently reduced the expression of TFs that at neural plate stages are enriched in either NP, NC or Epi (Figure 4). Ectopic expression of Six1 in the dorsal midline decreased the expression of several genes expressed in the early NP including *foxd4*, *sox2*, *sox3*, *irx1*, *irx2* and *irx3* (Figures 4A–C). Previous work indicated that Six1 promotes PPR fates by upregulating other PPR genes and downregulating the NC specifier *foxd3* (Brugmann et al., 2004). In concordance with those findings, we observed that ectopic expression of Six1 in NC progenitors reduced the expression of many TFs enriched in the NC domain at neural plate stages - *foxd3*, *sox9*, *msx1*, *pax3*, *tfap2* and *zic1-3* - in the majority of embryos (Figures 4D,E). Interestingly, a small percentage of embryos showed expansion of *zic2* and *zic3* expression in their NC and NP domains (Figures 4E,F). Ectopic expression of Six1 in the ventral ectoderm reduced expression of several TFs enriched in the Epi domain at neural plate stages (*foxi1, dlx3, dlx5, dlx6*; Figures 4G,H).

Dlx5 is a specifier of the dorso-lateral epidermis in *Xenopus* (Luo et al., 2001), and upregulates the epidermal genes *Gata3* and *foxi1* in chick, fish and frog (McLarren et al., 2003; Matsuo-Takasaki et al., 2005; Kwon et al., 2010; Pieper et al., 2012). Ectopic expression of Dlx5 frequently reduced the expression of TFs that at neural plate stages are enriched in either NP, NC or PPR (Figure 5). Dlx5 misexpression in the dorsal ectoderm reduced early NP expression of *foxd4* and *sox2* (Figure 5A); the latter result is consistent with a similar experiment in chick (McLarren et al., 2003). Dlx5 misexpression in the dorso-lateral region resulted in both reduced and expanded *foxd3* (Figures 5A–C), but only reduced *six1* (Figures 5A,D) expression. The latter result was surprising since previous work indicated that Dlx5 directly upregulates *Six1* in mouse (Sato et al., 2010) and is required for *six1* expression (Woda et al., 2003; reviewed in Grocott et al., 2012).

### 3.2 Reduced Expression at Gastrula Stages is Cell Autonomous Whereas at Neurula Stages it is Both Cell Autonomous and Non-autonomous

In analyzing the specimens presented above, we noticed that in some cases the reduction in gene expression was always cell autonomous, i.e., reduction was only observed in cells that also were marked by the lineage tracer (e.g., Figures 3A,E), whereas for others the target TF was reduced in both the cells carrying the lineage tracer (cell-autonomous) and in adjacent cells not labeled by the lineage tracer (e.g., Figures 2A,B). This suggested that for some genes the effects were strictly within the single cell that inherited the injected mRNA (βGal-positive), whereas for others signaling from that cell to nearby neighbors likely also was involved. For the frequency analyses presented in Figures 2–5, we only scored embryos in which the lineage tracer overlapped with the domain being analyzed. However, in most experimental batches there usually were a few embryos in which the lineage tracer did not overlap but was in proximity to the domain of interest, likely due to mistargeted injections at cleavage stages. When we screened these cases, we found that for genes that were analyzed at late gastrula stages (*foxd4*, *sox2*, *msx1*, *dlx5*), there were no cases, regardless of the injected mRNA, of non-autonomous reduction of expression; reduction was only observed in βGal-positive cells (Figure 6A; Table 1). In contrast, for genes that were analyzed at neurula stages (*foxd3*, *six1*, *dlx3*, *dlx5*, *dlx6*, *foxi1*), there often were cases of reduced expression distant from the lineage labeled cells (Figures 2A,B, 6B,C; Table 2); this occurred most frequently when *foxd4* or *msx1* mRNA was injected. These results suggest that as development progresses, signaling from cells ectopically expressing the neural plate stage domain-enriched TFs likely contributes to the segregation of these domains.

### 3.3 Single Cell RNAseq Analysis

These analyses demonstrate that TFs enriched in the four domains at neural plate stages reduce the expression of TFs characteristic of each of the other domains. For these effects to be direct, the different TFs need to be expressed in the same cell. In fact, transcriptomic screens indicate that the early ectodermal regions express overlapping sets of TFs (Plouhinec et al., 2014; Hintze et al., 2017; Plouhinec et al., 2017; Trevers et al., 2018) and antibody staining demonstrated that single cells in these regions co-express more than 1 TF characteristic of a neural plate stage domain (Roellig et al., 2017). To assess whether the TFs we analyzed would be able to directly repress each other within a single cell, we mined the available *Xenopus* single cell RNAseq dataset (Briggs et al., 2018) and evaluated the co-expression pattern of TFs to determine whether they would have the opportunity to interact within single cells.

From the stage 13 dataset, a stage at the end of gastrulation that exclusively showed cell autonomous effects in our ISH assays, we captured cells expressing a particular TF within tissues annotated by Briggs et al. (2018) as “celltype” clusters that correspond to neural plate, neural crest, placode or non-neural ectoderm domains by their overall transcriptome signature. For each TF captured from these combined domains we assessed the number of cells that co-expressed at least one other domain-enriched gene. Most cells within a TF dataset, except for the *foxd4* dataset, expressed only that TF (i.e., were single labeled for the selected TF), but a large number expressed two different TFs (Table 3). Of the *foxd4*-expressing cells, many also expressed *sox2* and several also expressed *zic2*. We detected only 1 *foxd4*-expressing cell that also expressed *msx1* or *dlx5* and none that also expressed *foxd3*, *six1* or *foxi1*. Thus, almost all *foxd4*-expressing cells only co-expressed TFs that also are enriched in the NP. Of the *sox2*-expressing cells, a large number also expressed *zic2*, and only a few also expressed *foxd4*, *msx1*, *six1*, *dlx5* or *foxi1*; none co-expressed *foxd3*. Thus, *sox2*-expressing cells mostly co-expressed TFs that also are enriched in NP and/or NC. Of the *msx1*-expressing cells, many also expressed *sox2* or *zic2*, and a small number also expressed *foxd4*, *six1*, *dlx5* or *foxi1*. Thus, *msx1*-expressing cells mostly co-expressed TFs characteristic of the NB (i.e., NC + PPR). At stage 13, only four cells in the neural crest cluster expressed *foxd3* and one of those also expressed *sox2;* we know from other studies that *foxd3* is only just beginning to be expressed, so this small number of *foxd3*-positive cells is not unexpected. Of the *zic2*-expressing cells, many also expressed *sox2* and a small number also expressed *msx1*, *six1* or *dlx5*; none co-expressed *foxd4, foxd3* or *foxi1*. This confirms that *zic2*-expressing cells mostly co-expressed TFs enriched in NP and/or NC. Of the *six1*-expressing cells, many also expressed *sox2* or *dlx5*, and a small number also expressed *msx1* or *zic2*; none co-expressed *foxd4, foxd3* or *foxi1*. Thus, *six1*-expressing cells mostly co-expressed TFs characteristic of the NB. Of the *dlx5*-expressing cells, many also expressed *sox2* or s*ix1,* and a small number also expressed *msx1*, *zic2* or *foxi1*; none co-expressed *foxd4* or *foxd3*. Thus, *dlx5*-expressing cells also mostly co-expressed TFs characteristic of the NB. Of the *foxi1*-expressing cells, a small number also expressed *sox2*, *msx1*, *zic2*, *six1* or *dlx5*; none co-expressed *foxd4* or *foxd3*. Thus, some *foxi1*-expressing cells mostly co-expressed NB genes. These data demonstrate that by the end of gastrulation, many of the cells identified as belonging to a particular ectodermal domain or “celltype” cluster by their overall transcriptomic signature (Briggs et al., 2018), co-express more than one domain-enriched TF. Thus, there is ample opportunity for repressive interactions between TFs within single cells at the end of gastrulation.

This analysis was repeated for stage 13 cells that expressed more than two of the selected domain-enriched TFs. We found that only a small number expressed three different TFs and rare cells expressed four different TFs (Table 4). For cells expressing three different TFs, we found that, independent of the TF dataset analyzed, particular combinations of 3 TFs predominated (color coded in Table 4): *sox2+zic2+foxd4* (orange, *n* = 12 cells), *sox2+msx1+zic2* (yellow, *n* = 48), *sox2+zic2+dlx5* (blue, *n* = 21), *sox2+six1+dlx5* (green, *n* = 22), *sox2+msx1+six1* (red, *n* = 3), and *msx1+six1+dlx5* (grey, *n* = 2). Overall, *sox2*-positive cells were most frequently co-expressed with other TFs. These data demonstrate that at the end of gastrulation, many cells express more than one domain-enriched TF and triple- and quadruple-labeled cells were present but not abundant. These single cell transcriptomic analyses confirm the bulk RNAseq study that reported that different pieces of ectoderm dissected at the end of gastrulation express TFs characteristic of more than one neural plate stage domain (Plouhinec et al., 2017). Our observation that by the end of gastrulation single cells rarely carry the transcriptional signature of all four domains is consistent with their report that the dissected domains have distinct transcriptional signatures by this stage. However, the combinations suggest a preferred domain combination: *sox2+zic2+foxd4* likely represents NP; *sox2+msx1+zic2* and *sox2+zic2+dlx5* likely represent the neural crest portion of the NB; *sox2+six1+dlx5*, *sox2+msx1+six1* and *msx1+six1+dlx5* likely represent the PPR portion of the NB. The quadruple labeled cell signatures were each consistent with an NB signature.

Since numerous cells in chick co-express TFs characteristic of more than one domain even as late as neural tube closure (Roellig et al., 2017), we asked if the same occurs in *Xenopus* by performing the single cell RNAseq analysis on the stage 18 (neural tube closure) dataset from Briggs et al. (2018). It should be noted that the complexity of the several selected “celltype” clusters within the SPRING plot for this stage made it difficult to eliminate the possibility of that some non-domain cells were included in the analysis. Nonetheless, for the most part the patterns of TF co-expression were similar to those observed at stage 13. Of the *foxd4*-expressing cells, many also expressed *sox2* or *zic2* and only a small number also expressed *msx1, foxd3*, *six1*, *dlx5,* or *foxi1*. This pattern was very similar to that of the stage 13 dataset in which most *foxd4*-expressing cells only co-expressed TFs that also are enriched in the NP. Of the *sox2*-expressing cells, many also expressed *zic2* or *msx1*, and a smaller number also expressed *foxd4*, *msx1*, *foxd3*, *six1*, *dlx5* or *foxi1*. Of the *msx1*-expressing cells, many also expressed *sox2*, *zic2* or *dlx5*, and a small number also expressed *foxd4*, *foxd3*, *six1* or *foxi1*. Of the *foxd3*-expressing cells, several also expressed *msx1* or *zic2*, a few also expressed *foxd4*, and a small number also expressed *sox2*, *six1*, *dlx5* or *foxi1*. Of the *zic2*-expressing cells, many also expressed *sox2* or *msx1,* and a small number also expressed *foxd4*, *foxd3*, *six1*, *dlx5*, or *foxi1*. Of the *six1*-expressing cells, many also expressed *sox2* or *dlx5*, and a small number also expressed *foxd4, msx1*, *foxd3*, *zic2* or *foxi1*. In each of these cases, the patterns of TF co-expression were similar to those observed at stage 13. However, the co-expression patterns of TFs that are Epi-enriched at neural plate stages were moderately different from stage 13. Of the *dlx5*-expressing cells, some also expressed *msx1* and a small number also expressed *foxd4, sox2*, *foxd3, zic2*, *six1* or *foxi1*; this is different from the stage 13 co-expression that was predominantly *sox2* or *six1*. Of the *foxi1*-expressing cells, most co-expressed *dlx5*, several co-expressed *sox2* or *msx1*, and a small number co-expressed *foxd4*, *zic2*, or *six1*; none co-expressed *foxd3*. This was a shift towards dlx5 co-expression compared to stage 13. When this analysis was extended to cells co-expressing three or more TFs, we found that many cells co-expressed three of the selected domain-enriched TFs (Table 6). The pattern of expression was more complex compared to stage 13, perhaps because there were many more cells and more complex “celltype” clusters in the dataset. However, like stage 13, particular combinations predominated, as color coded in Table 6: *foxd4*-expressing cells mostly co-expressed other NP-enriched genes; *sox2*-, *zic2*- and *msx1*-expressing cells predominantly co-expressed each other; *six1*-expressing cells mostly co-expressed *dlx5;* and very few *dlx5-* or *foxi1*-expressing cells co-expressed three or more TFs (Tables 5, 6). It also was rare for cells in these clusters to co-express 4 or 5 TFs (Table 7). Overall, these data indicate that even as late as neural tube closure, many of the cells identified as belonging to the four ectodermal domains by their overall transcriptomic signature (Briggs et al., 2018) co-express more than one domain-enriched TF, providing an opportunity for continued repressive interactions between TFs within single cells.

**TABLE 6:** Number of single cells co-expressing three domain-enriched transcription factors at stage 18.

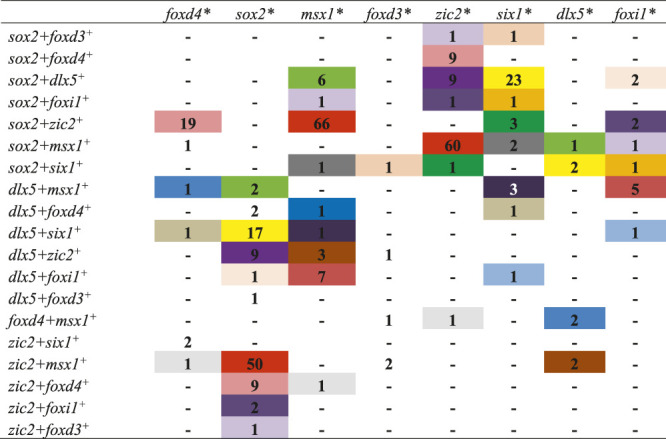	
	

Cells labeled with the same color co-expressed the same combination of three domain-enriched transcription factors.

*Transcription factor dataset that was queried.

^+^The transcription factors that were co-expressed with the factor at the top of each column.

**TABLE 7:** Number of single cells co-expressing four or more domain-enriched transcription factors at stage 18.

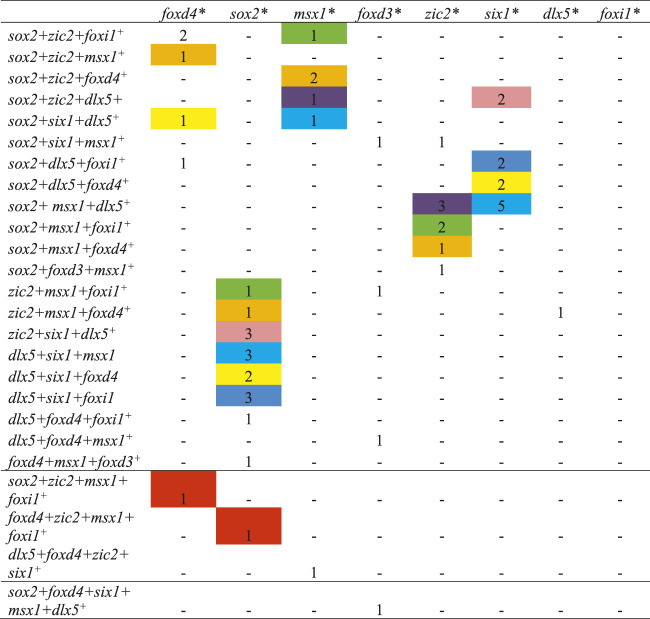

Cells labeled with the same color co-expressed the same combination of four or more domain-enriched transcription factors.

*Transcription factor dataset that was queried.

^+^The transcription factors that were co-expressed with the factor at the top of each column.

## 4 Discussion

It is well appreciated that the embryonic ectoderm becomes separated into neural and non-neural domains in response to signaling gradients of various growth factors, in particular BMP, Wnt and FGF (reviewed in Stuhlmiller and Garcia-Castro, 2012; Saint-Jeannet and Moody, 2014; Pla and Monsoro-Burq, 2018; Streit, 2018; Schlosser, 2021). By the time that the neural tube closes four domains - NP, NC, PPR and Epi—can be distinguished by a distinct suite of TFs that are thought to impose domain-specific identity (reviewed in Milet and Monsoro-Burq, 2012; Moody and LaMantia, 2015; Streit, 2018; Seal and Monsoro-Burq, 2020; Thawani and Groves, 2020). However, a number of studies have shown that the TFs that we used in our study as landmarks of these four domains are not exclusively expressed. Even as early as gastrulation, expression domains overlap and regions are broadly competent to give rise to other domains when transplanted (Schlosser and Ahrens, 2004; Pieper et al., 2012; reviewed in; Grocott et al., 2012; Schlosser, 2021). For example, Plouhinec et al. (2014), Plouhinec et al. (2017) reported that although dissected regions of gastrula ectoderm could be recognized by their overall transcriptomic signatures, genes considered highly specific for one region could be detected at lower levels of expression in adjacent regions. In addition, many TFs that are considered “domain-specific” at neurula stages are required at early stages for the formation of more than one domain, and at later stages participate in specifying the fate of a single domain. For example, using both loss- and gain-of function approaches, Maharana and Schlosser (2018) demonstrated that Zic1, Pax3, Hairy2b, TFap2, Msx1, Vent2 and Foxi1 each are required for the normal expression of an NC specifier (*foxd3*) and a PPR specifier (*six1*). Likewise, at early stages *msx1* is required in the NB for the expression of both NC and PPR genes, but at later stages promotes NC and represses PPR fates (Tribulo et al., 2003; Monsoro-Burq et al., 2005; Phillips et al., 2006).

In the present study, we examined whether the ectopic expression of a TF considered a specifier of one neural plate stage domain would alter the expression of TFs enriched in the other domains. We found that the expression of nearly every TF was reduced by the introduction of every other domain-enriched TF. One exception was the occasional expansion of *zic2* and *zic3* NC domains by Six1. This result is consistent with previous work. Maharana and Schlosser (2018) showed by knockdown experiments that Six1 is required for the NC expression of a related gene, *zic1*, and that Six1 overexpression expands *zic1*. Likewise, Brugmann et al. (2004) showed by knockdown that Six1 is required for *zic2* expression and in some cases over-expression expands the *zic2* domain. The other exception was the observed expansion of *foxd3* by Dlx5 in about a third of the cases. In *Xenopus*, Dlx5 is considered a specifier of dorso-lateral epidermis (Luo et al., 2001), but at early stages its expression domain overlaps the NB which contains NC progenitors. In chick, Dlx5 tends to downregulate *msx1* (McLarren et al., 2003; Stuhlmiller and Garcia-Castro, 2012) and in fish and frog Dlx family members upregulate *foxi1* (Matsuo-Takasaki et al., 2005; Kwon et al., 2010; Pieper et al., 2012). While some gene regulatory networks depict members of the Dlx family as promoting PPR fate and repressing NC fate (e.g., Grocott et al., 2012), others indicate that at early gastrula stages Dlx factors promote both PPR and NC genes (McLarren et al., 2003; Maharana and Schlosser, 2018). These exceptions point out that it will be important to experimentally discriminate between the early and later effects of each of these TFs in future experiments. Nonetheless, the overwhelmingly consistent observation that TFs enriched in one neural plate stage domain reduced the expression of TFs enriched in a different domain supports previous proposals (Schlosser, 2006; Grocott et al., 2012; Moody and LaMantia, 2015; Roellig et al., 2017) that mutual transcriptional repression between TFs contributes to the segregation of the four ectodermal domains.

### 4.1 Domain-Specifying Transcription Factors Act in a Mutually Repressive Manner

In order for one TF to reduce the expression of another TF they either are both expressed in the same cell and regulate each other’s expression in a cell autonomous manner, or they regulate downstream signaling pathways that affect gene expression in adjacent cells. In our analysis of lineage-labeled clones we found that at gastrula stages the effect of an ectopically expressed TF was exclusively cell autonomous, indicating that the mis-expressed TF repressed the target TF by acting within the same cell. It also suggests that individual cells normally express factors that are characteristic of more than one domain that interact transcriptionally to eventually result in a domain-specific fate. By analyzing a single cell RNAseq dataset of ectodermal clusters at the end of gastrulation, we indeed identified many cells that expressed TFs typical of more than one domain. These findings support the conclusions of several previous studies. Microarray analysis of precisely dissected ectodermal domains from chick showed that PPR gene clusters expressed many NP-enriched and NC-enriched genes (Hintze et al., 2017). RNAseq analysis of similarly dissected *Xenopus* domains showed that while transcriptomic signatures could be discerned for the various domains as early as late gastrula, the neural border tissue expressed TFs characteristic of more than one domain (Plouhinec et al., 2017). At the single cell level using antibody staining for TF proteins, Roellig et al. (2017) reported that about 50% of NB cells co-expressed two different “domain-specific” TFs and about 7% expressed three markers. These authors also found that Sox2, designated an NP TF, and Pax7, designated an NC TF, were mutually repressive within single cells. Interestingly, other studies noted that the Roellig et al. (2017) data showed a preference among the NB progenitors for expressing primarily NP + NC markers, suggestive of the binary competence model (Maharana and Schlosser, 2018; Pla and Monsoro-Burq, 2018). Our analysis of the single cell RNAseq data of Briggs et al. (2018) showed a similar preference among both stage 13 and stage 18 clusters to express NP + NC markers (Tables 3–7). In accord with the results from Roellig et al. (2017), we also find single cells at neural tube closure stages that continue to express multiple domain-enriched TFs. Together, these results support the proposed model in which individual ectodermal cells are initially multipotent (Grocott et al., 2012; Hintze et al., 2017; Roellig et al., 2017; Trevers et al., 2018); individual cells express TFs that over time repress each other to subsequently determine a cell’s domain-specific fate by restricting their transcriptomic signature.

### 4.2 Cell-to-Cell Signaling Contributes to Domain Separation by Neurula Stages

In the embryo as well as in organoids, boundaries form between different progenitor fields as cells acquire different regional, tissue and functional fates. Boundary formation is documented to involve interactions between adjacent fields that include differential transcriptional programs, position within a morphogen gradient, local cell-cell interactions and highly regulated cell rearrangements (Irvine and Rauskolb, 2001; Dahmann et al., 2011; Jaeger, 2011; Martyn and Gartner, 2021). Many studies have demonstrated that progenitor cells and gene expression territories characteristic of the four ectodermal domains initially overlap and gradually segregate in response to local interactions assumed to be at the boundaries (reviewed in Moody and Saint-Jeannet, 2014; Saint-Jeannet and Moody, 2014; Pla and Monsoro-Burq, 2018; Schlosser, 2021). In concordance, we observed that TFs enriched in one neural plate stage domain reduced the expression of TFs enriched in the adjacent domains. For example, NC-enriched TFs reduced the expression of both NP-enriched genes and PPR-enriched genes, and PPR-enriched TFs reduced the expression of both NC-enriched genes and Epi-enriched genes. However, we also observed this effect after ectopic expression of a TF in a non-adjacent domain, for example, an NP-enriched gene mis-expressed in the PPR or Epi. By methodically expressing a domain-enriched TF in each of the major precursors of each of the other domains, we found that in every case TFs of both adjacent and non-adjacent domains caused mutual repression. This indicates that the interactions that segregate NP, NC, PPR and Epi domains are not confined to local interactions at boundaries.

There are several comprehensive reviews of the multiple studies that demonstrate both local and distant signaling that regulate the formation of the four ectodermal domains (Grocott et al., 2012; Milet and Monsoro-Burq, 2012; Stuhlmiller and Garcia-Castro, 2012; Saint-Jeannet and Moody, 2014; Schlosser, 2014; Pla and Monsoro-Burq, 2018; Streit, 2018; Schlosser, 2021). Inductive signals can be transmitted through the plane of the ectoderm and from underlying mesoderm and pharyngeal endoderm (Papalopulu and Kintner, 1993; Woda et al., 2003; Ahrens and Schlosser, 2005; Litsiou et al., 2005; Pieper et al., 2012; Watanabe et al., 2015; Hintze et al., 2017; Trevers et al., 2017). Our ISH analyses indicate that at gastrulation stages cell-cell signaling plays little role in transcriptional repression within an ectodermal domain; changes in gene expression were limited exclusively to cells carrying the lineage label. However, while clones expressing ectopic TFs at neurula stages also exhibited a predominance of cell autonomous reduced expression, we also observed repression in cells adjacent to, but not overlapping with, the lineage-labeled cells. This observation suggests that the mis-expressed TF also repressed the target TF indirectly *via* cell-to-cell signaling. While there are several examples of cell-cell signaling being important in placode and neural crest induction (Begbie et al., 1999; Brugmann et al., 2004; Ahrens and Schlosser, 2005; Litsiou et al., 2005; Monsoro-Burq et al., 2005; Watanabe et al., 2015; Hintze et al., 2017; Plouhinec et al., 2017; reviewed in; Milet and Monsoro-Burq, 2012; Stuhlmiller and Garcia-Castro, 2012; Saint-Jeannet and Moody, 2014; Pla and Monsoro-Burq, 2018; Streit, 2018; Schlosser, 2021), there also is evidence for indirect signaling. For example, Dlx5 indirectly induces epidermal and PPR genes (McLarren et al., 2003) and Zic1 affects PPR gene expression at a distance by regulating retinoic acid signaling (Jaurena et al., 2015; Dubey et al., 2021). Since an alternate explanation is that the *nβgal* mRNA was selectively diluted in part of the clone, it will be important to confirm our lineage tracing data by grafting TF-expressing cells into an ectopic domain and observing reduced expression in the adjacent host tissue, as has been elegantly shown for *dlx5* and *six1* in *Xenopus* (Woda et al., 2003; Ahrens and Schlosser, 2005). If such future experiments support the involvement of cell-cell signaling initiated by the TFs studied in this work, it will be important to determine whether the signals originate within the plane of the ectoderm or from underlying tissues.

### 4.3 Domain Separation is Gradual

Many different experimental approaches indicate that the separation of the four ectodermal domains is a gradual process. For example, a microarray analysis of a large number of genes expressed by PPR explants proposed that head mesoderm induces a “pre-neural” state that expresses a few TFs that then induce a “PPR-primed state” that expresses genes that next induce PPR specifier genes (Hintze et al., 2017). A transcriptomic study of the developmental timing of gene expression in the chick epiblast indicated that at pre-primitive streak stages this tissue is already specified to a neural plate border state (Trevers et al., 2018). A comprehensive gain- and loss-of-function analysis showed that dorsal ectoderm TFs (*zic1-5*, *sox3*) and ventral ectoderm TFs (*dlx3/5*, *gata2/3*, *vent1/2*, *foxi1/3*, *msx1*) broadly overlap in an intermediate zone, and this overlap deceases over development until boundaries are formed. Principal component analysis of the transcriptomes of dissected *Xenopus* ectodermal regions revealed distinct domains at gastrula stages that resolved as development proceeded (Plouhinec et al., 2017). Our data also indicate that transcriptional interactions that specify the fate of a domain begin as early as gastrulation stages; by mid-gastrula NC, PPR and Epi factors reduced the expression of NP factors (*foxd4*, *sox2*) and NC and PPR factors reduced Epi factors (*dlx5*, *foxi1*, *epiker*). Roellig et al. (2017) analyzed protein rather than transcript levels and also found single cells expressing more than one domain-typical TF protein as early as gastrula and as late as neural fold closure. Although they did not provide the spatial distribution of these cells, the authors noted that double- and triple-labeled Six1-positive cells predominated in the lateral side of the border zone, which is where the PPR will form. They also quantitatively mapped the protein expression domains of four domain-enriched TFs, albeit not at the single cell level, and also found evidence of some regionalization of expression. The NP domain highly expressed Sox2 protein but not the other TFs; the border zone adjacent to the NP expressed moderate levels of Sox2, Pax7 and Tfap2a; the middle region of the border zone expressed high levels of Pax7 and Tfap2a and lower levels of Sox2; the lateral region of the border zone expressed low levels of Sox2 and Pax7 and moderate levels of Tfap2a and Six1; and only Tfap2a and Six1 were expressed in the most lateral region analyzed. It would be most interesting, when specific antibodies for the TFs analyzed in our study are available in *Xenopus*, to use a similar approach to determine whether there is any spatial restriction of cells expressing single or multiple TFs as predicted by the scRNAseq data.

### 4.4 Conclusion

Together, several previous studies and the data presented herein provide overwhelming evidence that the segregation of the four embryonic ectodermal domains begins during gastrulation. We found that at this stage it is mediated primarily by direct repressive interactions between TFs expressed within individual cells, but by late neural plate stages indirect interactions with adjacent cells assists in establishing boundaries and driving ultimate domain-specific fate decisions. Several future experiments are needed to more fully understand the molecular regulation of these processes, such as identifying: 1) stage- and domain-specific enhancers; 2) the TFs bound to them; and 3) the identity of and tissue source of the non-autonomous signals initiated by these TFs. With this information a more complete gene regulatory network can be constructed and utilized to predict dysmorphologies that may arise due to subtle changes in gene expression and interactions.

## Data Availability

The datasets presented in this study can be found in the paper and in Briggs et al. (2018).
